# Neurogenesis in Response to Synthetic Retinoids at Different Temporal Scales

**DOI:** 10.1007/s12035-017-0440-7

**Published:** 2017-02-27

**Authors:** Hesham Haffez, Thabat Khatib, Peter McCaffery, Stefan Przyborski, Christopher Redfern, Andrew Whiting

**Affiliations:** 1 Department of Chemistry, Centre for Sustainable Chemical Processes, Durham University, South Road, Durham, UK; 2 Department of Biosciences, Durham University, South Road, Durham, DH1 3LE UK; 3 Northern Institute for Cancer Research, Medical School, Newcastle University, Newcastle upon Tyne, NE2 4HH UK; 4 Department of Biochemistry and Molecular Biology, Pharmacy College Helwan University, Cairo, Egypt; 5 School of Medicine, Medical Sciences and Nutrition, Institute of Medical Sciences, Foresterhill, Aberdeen, AB25 2ZD UK

**Keywords:** Retinoids, Retinoic acid, Neurogenesis, Neuroblastoma, Stem cells, Gene expression

## Abstract

All-trans retinoic acid (ATRA) plays key roles in neurogenesis mediated by retinoic acid receptors (RARs). RARs are important targets for the therapeutic regulation of neurogenesis but effective drug development depends on modelling-based strategies to design high-specificity ligands in combination with good biological assays to discriminate between target-specificity and off-target effects. Using neuronal differentiation as a model, the aim of this study was to test the hypothesis that responses across different temporal scales and assay platforms can be used as comparable measures of retinoid activity. In biological assays based on cell phenotype or behaviour, two structurally similar synthetic retinoids, differing in RAR affinity and specificity, retained their relative activities across different temporal scales. In contrast, assays based on the transcriptional activation of specific genes in their normal genomic context were less concordant with biological assays. Gene-induction assays for retinoid activity as modulators of neurogenesis require careful interpretation in the light of variation in ligand-receptor affinity, receptor expression and gene function. A better characterization of neuronal phenotypes and their regulation by retinoids is badly needed as a framework for understanding how to regulate neuronal development.

## Introduction

Retinoic acid (RA) receptor signalling plays key roles in cell and tissue patterning, neurogenesis and homeostasis, both directly via nuclear retinoic acid receptors (RARs) and indirectly by interactions with other ligand-dependent signalling mechanisms via shared co-factors, receptor partners and ligand cross-talk [1, 2]. This signalling diversity underlies the potential of RA and related compounds as important drugs for medicinal use, ranging from cancer therapeutics to novel treatments for diseases associated with ageing and neuronal health. In normal cellular and tissue development, intracellular levels of the main biologically active RA isomer, all-trans RA (ATRA), are finely regulated by conversion from vitamin A (retinol), via cellular binding proteins which mediate the transfer of retinol to retinol dehydrogenases for conversion to ATRA. The transfer of ATRA to RARs is mediated by cellular retinoic acid binding proteins (CRABPs) to achieve transcriptional regulation [3, 4] as part of normal cellular homeostasis, and to drive cell and tissue patterning during embryogenesis and tissue differentiation [1, 4, 5].

RARs are encoded by the transcripts of three separate genes, RARA (RAR-α), RARB (RAR-β) and RARG (RAR-γ), and specificity in responses at a cellular level are driven by tissue- and stage-specific variation in gene expression. This is also coupled with variation of splicing patterns to generate N-terminal variants [6] facilitating combinatorial interactions with different transcriptional coregulators [7]. Temporal regulation of gene expression is facilitated by ATRA degradation mediated by specific cytochrome P450 enzymes which are themselves regulated by ATRA [8, 9]. One consequence of this finely tuned metabolism is that ATRA has a short lifetime when added to cells or used therapeutically in vivo [10, 11]. Thus, although the spatial and temporal regulation of ATRA synthesis and delivery to RARs provides exquisite control of ATRA-dependent gene expression, this also provides significant challenges for the development of drugs to regulate ATRA signalling for clinical benefit. The key requirements for such drugs will be stability, so that ligand concentrations can be maintained in relevant cells and tissues, and providing sufficient RAR specificity to target particular processes, tissues or cell types.

There has been considerable progress in designing retinoid-like molecules which are considerably more stable than ATRA in intra- and extra-cellular environments [12]. To be classed as a retinoid, compounds should produce cellular effects by specific interactions with the ligand binding domain of the RARs. Recent modelling studies have shown that, despite the high degree of sequence conservation, there are important differences between receptor types in the shape of the ligand binding pocket [13]; this has implications for the design of modified stable synthetic retinoids for targeting specific biological processes. Furthermore, it is not just necessary to ensure good fit of the synthetic retinoid into the ligand binding domain (LBD) of RARs but also to ensure that the ligand-LBD complex has sufficient structural integrity to ensure effective coactivator recruitment for transcriptional regulation [14, 15].

**Fig. 1:**
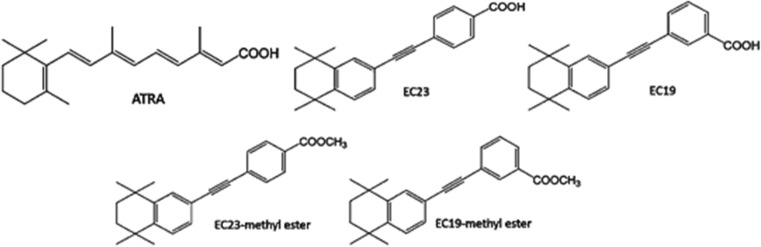
Chemical structures of ATRA and the synthetic analogues, EC23, EC19 and methyl esters. [9]

ATRA has an important role in neurogenesis [17], and we have developed the synthetic retinoids 4-(5,5,8,8-tetramethyl-5,6,7,8-tetrahydronaphthalen-2-ylethynyl)benzoic acid (para-isomer; EC23) and 3-(5,5,8,8-tetramethyl-5,6,7,8-tetrahydronaphthalen-2-ylethynyl)benzoic acid (meta-isomer; EC19) [18] as tools for studying neurogenesis in vitro (Fig. 1). These compounds are chemically and biologically more stable than ATRA; receptor binding and molecular docking studies show that EC23 binds to all three RARs in a manner similar to ATRA whereas EC19 has fewer interactions between key residues in the RAR-α and RAR-γ binding pockets while being a better fit to the larger binding pocket of RAR-β [13]. However, biological models are essential for assessing novel retinoids as potential clinical drugs or experimental tools and to discriminate between RAR-dependent activity and non-specific or downstream effects. In addition, ATRA may have distinct concentration-dependent effects in promoting alternative differentiation pathways [16] and for cellular homeostasis [17]. Temporal scale in biological models is of critical importance, and many assays of retinoid activity are carried out at long timescales which may make results hard to reconcile with known RAR specificity.

The aim of this study was to test the hypothesis that short- and long-term responses can be used as equivalent measures of retinoid activity and metabolic stability in assays of synthetic retinoids in neurogenesis models. Neurogenesis and gene expression at different temporal scales were compared in TERA2.cl.SP12 pluripotent stem cells and SH-SY5Y neuroblastoma cells in response to EC19 and EC23, and their methyl esters, using ATRA as the positive control. The methyl esters were included for some assays because although they show reduced RAR binding activity as a result of the absence of the key carboxylic acid-arginine residue interaction [13], esters may be relevant for biological studies if the parent compounds are released by endogenous esterase activity.

## Material and Methods

### Retinoid Solutions

Stock solutions of synthetic retinoids EC19 and EC23 and their methyl esters were prepared as reported earlier [18]; ATRA was from (Sigma-Aldrich, Poole, UK). All compounds were dissolved in DMSO (Sigma-Aldrich) to 10 mM. Aliquot stock solutions were stored at −20 °C in the dark.

### Cell Culture

Human pluripotent TERA2.cl.SP12 embryonal carcinoma stem cells were cultured [19], under low-light conditions to minimize retinoid isomerisation, in Dulbecco’s modification of Eagle’s medium (DMEM; Sigma-Aldrich) supplemented with 10% FCS (Gibco), 2 mM L-glutamine and 100 units each of penicillin and streptomycin (Gibco). Cultures were passaged using acid-washed glass beads unless a single-cell suspension was required for counting, in which case, a 0.25% trypsin/EDTA (Lonza) solution was used. Human SH-SYSY neuroblastoma cells were cultured in DMEM F12/Ham (1:1) containing 2 mM L-glutamine, supplemented with 10% FCS at 37 °C with 5% CO_2_ in air [20]. Cell suspensions were obtained by treating adherent cells with 1 ml sterile PBS and incubation at 37 °C for 3–5 min. Culture media were replaced every 3–4 days.

### Flow Cytometry

Flow cytometry was carried out on live TERA2.cl.SP12 cells, incubated at a density of 0.2 × 10^6^ cells per 25-cm^2^ flask for 12–24 h before treatment with 10 μM retinoid for 7 days. Specific cell-surface primary antibodies were used: SSEA-3 (1:10), (University of Iowa Hybridoma Bank), TRA-1-60 (1:50), (Abcam) and neural cell marker A2B5 (1:40), (R&D Systems). Cell suspensions were centrifuged at 1000 rpm and resuspended in wash buffer (0.1% BSA in PBS) and added to 96-well plate for incubation with the primary antibodies, followed by several washes and then incubation with FITC-conjugated secondary antibody IgM (1:128) (Sigma-Aldrich). Labelled cells were analysed in a GuaveEasyCytePlus System (Millipore) flow cytometer and thresholds determining the numbers of positively expressing cells were set against the negative control antibody, P3X, a generous gift from Prof. P. Andrews, Sheffield University.

### Gene Expression Analysis

Real-time quantitative PCR (qPCR) was carried out on cell lysates immediately after detachment with 0.25% trypsin–EDTA. Cells were seeded at a density of 1 × 10^6^ cells per 25 cm^2^ flask (BD falcon) 12–24 h before retinoid treatment. Commercial RNA extraction (Qiagen) and reverse transcription (Applied Biosystems) kits were purchased and procedures used according to manufacturer instructions. Real-time qPCR was performed using the TaqMan® Universal PCR master Mix (Life technologies) and TaqMan® gene expression system (Applied Biosystems) based on probe sets to the specific genes to be analysed: RAR-β (Hs00233407_m1), CYP26A1-A1 (Hs00175627_m1), RAR-α (Hs00940448_g1), RAR-γ (Hs01559234_m1), PAX-6 (Hs01088112_m1), NeuroD1 (Hs01922995_s1). GADPH (Hs02758991_g1) and ACTB (Hs99999903_m1) were used as internal control genes for TERA2.cl.SP12 and SH-SY5Y cells, respectively.

### Immunocytochemistry

TERA-2.cl.SP12 cells were seeded at 5000 cells per well on poly-d-lysine (25 μg/ml) coated cover slips 22 × 22 mm, high precision (170 ± 5 μm) in 6-well plates. At the end of the experiment, cells were fixed in 4% para-formaldehyde (PFA) in PBS for 30 min at room temperature and rinsed with PBS. For intracellular staining, cells were permeabilised with 1% Triton-X-100 (Sigma) in PBS for 10 min at room temperature. Non-specific labelling was blocked by incubation for 1 h at room temperature with 1% goat serum (Sigma) in PBS with 0.2% Tween-20 (Sigma). Primary antibodies were diluted in blocking solution and incubated with cells for 1 h at room temperature with a β-III tubulin antibody (TUJ-1) 1:200 (Affymetrix eBioscience) or CK-8 antibody 1:500 (Affymetrix eBioscience). After washing with PBS, cells were incubated for 1 h in the dark with anti-mouse FITC-conjugated secondary antibody IgM 1:128 (Sigma) for A2B5 staining or anti-mouse Alexafluor 488 IgG 1:600 (Invitrogen) for TUJ-1 and CK-8 staining. Hoechst 33,342 nuclear staining dye (Molecular Probes) was used at 1:1000 in blocking solution after the secondary antibody step. Fixed and stained cells were visualized using a Leica SP5CLSM FLIM FCCS confocal microscope.

### X-gal Bioassay

Sil-15 cells (F9-RARE-lacZ cells) [21] were plated in a 0.1% gelatin-coated 96-well plate and grown to about 85–90% confluence in DMEM containing 10% foetal calf serum (Invitrogen/Gibco) and 0.8 mg/ml G418 sulphate (Sigma) for selection. Cells were washed twice with PBS, fixed with 100 μl per well of 1% glutaraldehyde for 15 min, washed again twice with PBS and β-galactosidase activity was visualized with 100 μl of a freshly prepared X-Gal developing solution (5-bromo-4-chloro-3-indolyl-β-d-galactopyranoside) added to each well. Colour was read on an Emax microplate reader at 650 nm.

### Neurite Outgrowth

SH-SY5Y cells were fixed and stained for TUJ-1 (1:1000; Sigma) after retinoid treatment for 5 days. For each neurite outgrowth experiment, 3 cover slips (in 3 wells) were used. The numbers of neurites were counted and traced for length measurement using a semi-automatic NeuronJ plugin for ImageJ software in each of 10 randomly-selected images for each cover slip. Average neurite length was calculated by dividing the total neurite length by the total number of neurites per image.

## Results

### Short-Term Responses to Retinoids: SH-SY5Y Cells

#### Retinoid-Induced Gene Expression

**Fig. 2:**
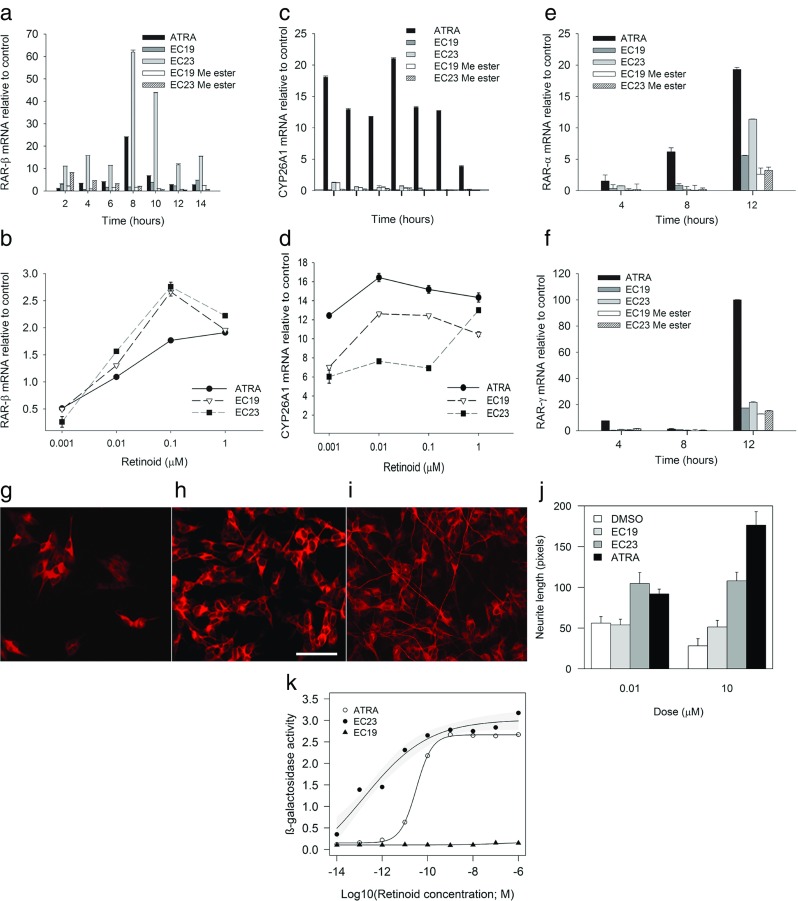
Short-term assay of retinoid properties in SH-SY5Y cells and Sil-15 reporter cells. **a**–**f** Real-time quantitative PCR (qPCR) analysis of mRNA expression for RAR-β (**a** time course, **b** dose-response), CYP26A1 (**c** time course, **d** dose-response), RAR-α (**e** time course) and RAR-γ (**f** time course) in SH-SY5Y cells treated with (*left* to *right*) ATRA, EC19, EC23 and methyl ester analogues. Dose-response experiments were performed with retinoid concentrations of 1, 0.1, 0.01 and 0.001 μM for 8 h, and time course experiments with 10 μM for up to 12 h. Quantification of target mRNA was relative to SH-SY5Y cells cultured with DMSO vehicle for the relevant time period and normalized to the internal reference gene (ACTB). Data represent mean ± SEM, *n* = 3. Neurite outgrowth by SH-SY5Y cells in response to control vehicle or EC19 or EC23 (10 μM each) is shown in the images (**g**–*i*), stained with the anti-beta III tubulin antibody TUJ-1 (*scale bar* 50 μm) (**g** control, **h** EC19, **i** EC23) and (**j**) bar graph for mean neurite length (pixels ± SEM, *n* = 3). There was a significant induction of neurite outgrowth in response to EC23 (ANOVA, *P* < 0.001) but not EC19, and no effect of dose at the concentrations used. The response by Sil-15 reporter cells to increasing concentrations of ATRA, EC19 or EC23 is shown in **k** where *grey shading* along the fitted curves defines the 95% confidence limits for the EC23 and EC19 data

The induction of RAR-β or CYP26A1 transcription is well established as a marker of retinoid-response [22]; therefore, the expression of these genes was tested with respect to retinoid dose and time of treatment, in addition to investigating retinoid-induced changes in expression of RAR-α and RAR-γ. In SH-SY5Y cells, RAR-β expression in response to 10 μM ATRA or EC23 increased to a maximum at 6 h, with EC23 showing greater activity. In contrast, EC19 and the methyl ester showed no, or minimal, activity at 10 μM (Fig. 2a). With respect to the induction of CYP26A1, only ATRA had good activity, with a rapid and sustained induction from 2 to 10 h (Fig. 2c). In dose-response experiments from 1 to 0.001 μM, EC19 and EC23 had similar peak activities for induction of RAR-β at 0.1 μM which exceeded that of ATRA by about 30% (Fig. 2b). Conversely, for CYP26A1 induction, although the synthetic retinoids were not inactive, ATRA showed consistently higher levels of activity over the whole dose range compared to the synthetic retinoids; indeed, for the latter, EC19 had greater activity than EC23, except at 1 μM (Fig. 2d).

As in some other cell types [23], ATRA induced RAR-α expression, but over a longer timescale than for RAR-β. In contrast to their induction of RAR-β, the synthetic retinoids were less effective at inducing RAR-α compared to ATRA; however, EC23 was at least twice as active as EC19 at the 12-h timepoint. Substantial induction of RAR-γ was only apparent after 12 h with ATRA and at this time EC19 and EC23 were only marginally more effective than their methyl esters (Fig. 2e, f).

#### Neurite Length

SH-SY5Y cells responded morphologically to retinoids with time-dependent increases in neurite length, with the greatest differential responses between the retinoids after 4 days or more [24]. At 0.01 and 10 μM, EC19 had no neurite-inducing capacity compared with the control vehicle (DMSO), unlike EC23, which had similar activity to ATRA at 0.01 μM (Fig. 2j); however, in contrast to ATRA which gave a dose-dependent increase in neurite length, the response to EC23 appeared saturated at 0.01 μM. Neurite extension is a lower-resolution technique over a more-extended timescale than the gene-expression assays, and there were clear differences between these assays in the relative activities of all retinoids at 10 μM (Fig. 2j).

#### X-Gal Reporter Bioassay Analysis

The relative transcriptional potency of the retinoids was also tested on Sil-15 reporter cells, which are F9 murine teratocarcinoma cells stably transfected with a LacZ gene under the control of a RA response element (RARE) promoter [21]. β-Galactosidase activity in these cells was assayed over retinoid concentration ranges of 10^−6^ M to 10^−14^ M over 24 h. EC23 was effective over the entire range, giving a 50% response between 10^−11^ to 10^−12^ M, compared to the 50% response of ATRA at 5 × 10^−10^ M. In contrast, EC19 showed very little activity (Fig. 2k).

### Longer-Term Responses to Retinoids: TERA-2. cl.SP12 Cells

#### Cell Differentiation Markers

**Fig. 3:**
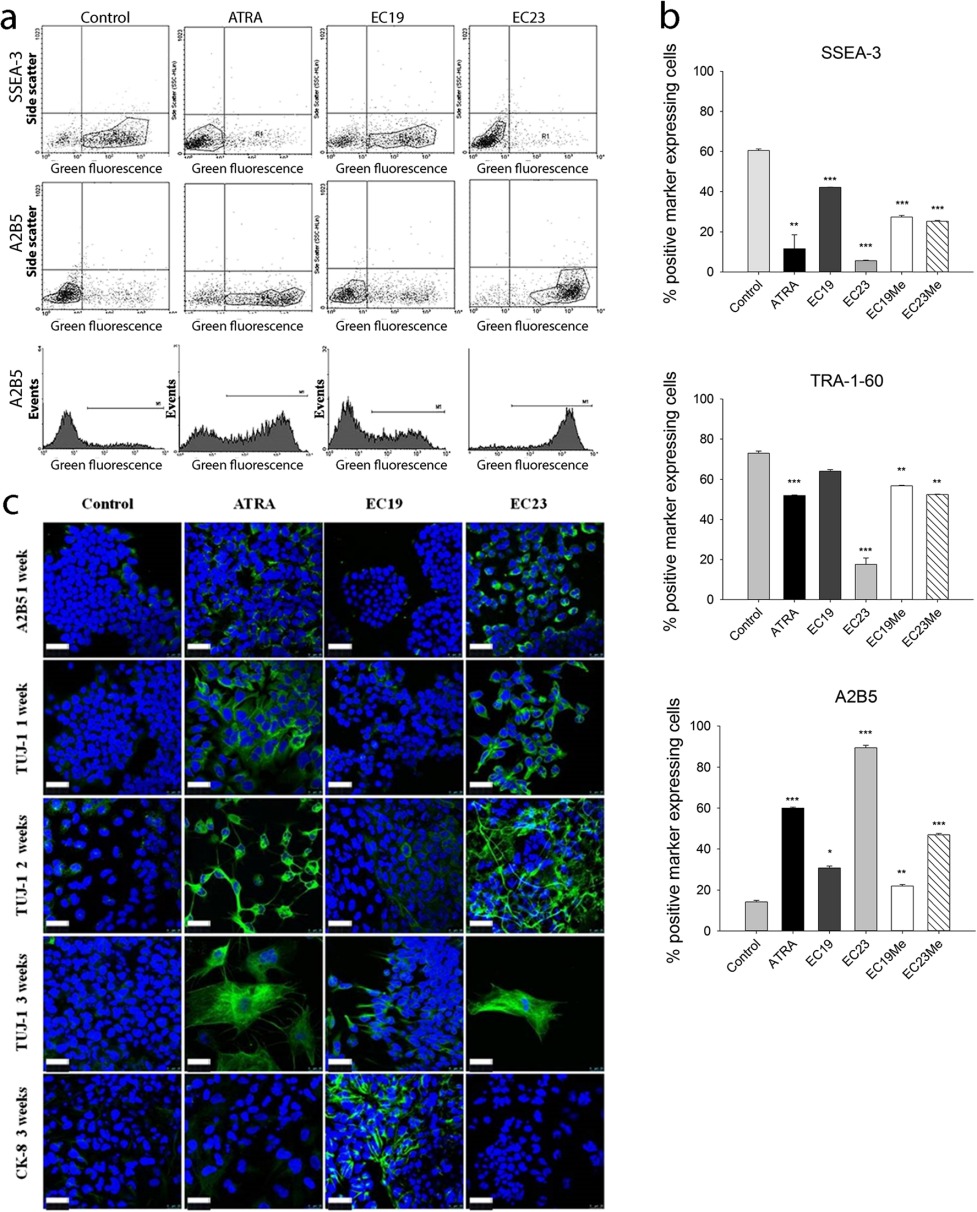
Longer-term assay of retinoid responses using the cell differentiation model of TERA2.cl.SP12 stem cells treated with 10 μM ATRA, EC19, EC23 or their methyl esters after 7 days. Control TERA2.cl.SP12 cultures were treated with 1% DMSO vehicle. **a** Flow cytometry analysis showing 2D plots of gated cells expressing SSEA-3 and A2B5 after retinoid treatment. **b** Histograms of percentage positive cells for the stem cell-surface markers SSEA-3, TRA-1-60 and the early stage neuronal marker A2B5. Results are presented as ±SEM, *n* = 3; *P* values for comaprisons with control are: *P < 0.05; **P < 0.01; ***P < 0.001. **c** Photomicrographs of TERA2.cl.SP12 cells stained for the neuronal (A2B5 and TUJ-1) and epithelial (CK-8) proteins after exposure to 10 μM ATRA, EC19 or EC23 for 1, 2 and 3 weeks. *Scale bar*, 25 μm

ATRA-induced differentiation of TERA2.cl.SP12 cells is characterized by the downregulation of the glycolipid antigen SSEA3 and the keratan sulphate-related antigen TRA-1-60, and the upregulation of the c-series ganglioside-specific antigen A2B5, characteristic of neuronal and glial cells [19]. TERA2.cl.SP12 cells were treated with 10 μM of each retinoid for 7 days and analysed for the expression of SSEA3, TRA-1-60 and A2B5 by flow cytometry. Control cultures, untreated or treated with the DMSO vehicle alone, showed high expression levels of SSEA-3 and TRA-1-60 with 60–70% of cells expressing these markers, but less than 20% of these cells expressing the neuronal differentiation marker A2B5 (Fig. 3a, b). After treatment with either 10 μM ATRA or EC23 for 7 days, there was a significant reduction in expression of SSEA-3 (20% of cells) and TRA-1-60 (50% of cells) and an induction of expression of A2B5, indicating a shift from a pluripotent state towards neuronal differentiation which was particularly marked with EC23; conversely, EC19 was less effective (Fig. 3a, b). The methyl ester of EC23 was less active than the parent compound, while the EC19 methyl ester was as active, or slightly more so, than EC19 (Fig. 3b).

Cell morphology and phenotypic fate within TERA2.cl.SP12 cell cultures were assessed by immunocytochemistry for neuronal markers (cell-surface A2B5; cytoplasmic βIII-tubulin, TUJ-1 antibody), and the epithelial marker cytokeratin 8 (CK-8). Control cultures (DMSO vehicle) showed low expression of all markers. After exposure to ATRA or EC23 for 7 days, expression of the neuronal markers substantially increased, in contrast to CK-8. After 14 and 21 days, βIII-tubulin expression increased further with the formation of more mature, differentiated neuronal cells where staining was localized to the cytoplasm and neuronal processes. In contrast, EC19 did not induce any substantial increase in expression of A2B5 or βIII-tubulin after 7 days, with low levels of βIII-tubulin-positive cells even after 21 days. However, the expression of CK-8 increased in EC19-treated cultures after 21 days, suggesting differentiation towards an epithelial phenotype (Fig. 3c).

#### Expression of Neuronal Lineage Marker Transcripts

**Fig. 4:**
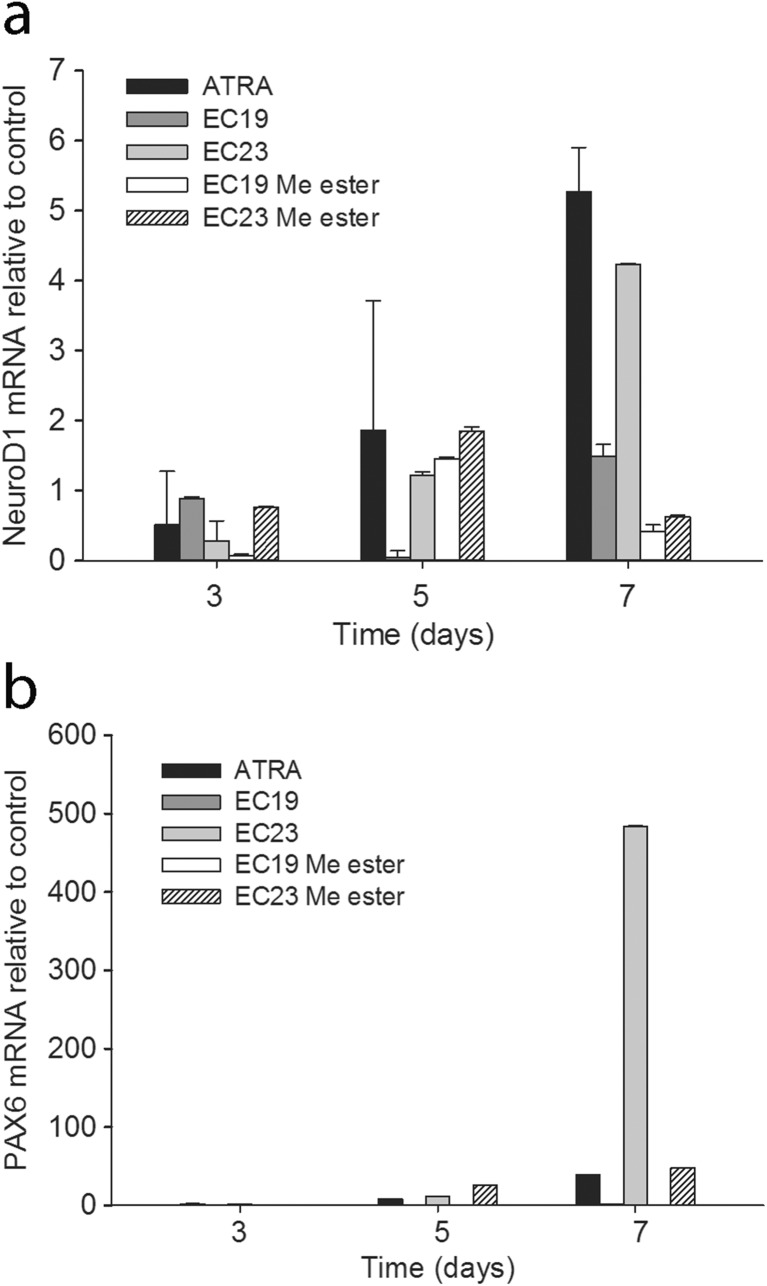
NeuroD1 (**a**) and PAX6 (**b**) gene expression in TERA2.cl.SP12 stem cells treated with 10 μM of ATRA, EC19, EC23 or their methyl ester analogues for 3, 5 and 7 days. Quantification is relative to TERA2.cl.SP12 cells cultured with 0.1% DMSO vehicle for 7 days and all data (mean ± SEM, *n* = 3) were normalized to the internal reference gene (GADPH)

The neuronal markers NeuroD1 and PAX-6 are predominantly expressed in the nervous system, particularly later in development [25, 26]. NeuroD1 was upregulated in TERA2.cl.SP12 cells showing a linear time-dependent increase in response to 10 μM ATRA or EC23; EC19 and the methyl esters of both synthetic retinoids had much lower activities (Fig. 4a). PAX6 was substantially upregulated only after 7 days of treatment and EC23 was 10 times more effective than ATRA with very little activity shown by EC19 (Fig. 4b).

## Discussion

With respect to the parent compounds, there were generally concordant responses, summarized by rank in Table 1, between long-term and short-term biological response assays (differentiation, neurite extension, reporter assays) where both EC23 and EC19 maintained their relative activities over different temporal scales. The methyl esters of EC23 and EC19 usually had low, or intermediate, activity. Although structural studies and ligand binding/activity assays suggest that these methyl esters may have direct activity as RAR ligands in their own right [13], esterase activity [27] releasing the free parent carboxylic acids may also contribute to biological activity. In contrast to biological response assays, assays of specific gene transcripts, whether as markers of neural differentiation status as with the NeuroD1 and PAX6 transcripts, or of short-term responses to retinoids such as the induction of expression of RARs or CYP26A1, were not directly comparable with the broader biological responses. This was particularly true for EC19 which, as predicted from structural studies, had activity in some gene-response assays but low activity in biological response assays. These results highlight two key factors in responses to retinoids: the dynamic mechanisms of individual gene regulation by retinoids and the retinoid mechanisms directing biological responses.

**Table 1:** Summary of ranked responses to retinoids in different assays. Retinoids are listed in rank order of ligand binding assay activity which is broadly concordant with molecular docking studies [13]; numbers represent rank order (1 = highest activity; 0 = no detectable activity) in different assays

	Short-term								Longer-term				
	Gene expression						Biological		Differentiation markers			Neuronal lineage	
Retinoid	RAR-β		cyp26		RAR-α	RAR-γ	Neurites	βgal	SSEA3	TRA-1-60	A2B5	NeuroD1	PAX6
	Peak	Dose	Peak	Dose			Peak						
EC23	1	1	2	3	2	2	2	1	1	1	1	2	1
ATRA	2	3	1	1	1	1	1	2	2	2	2	1	3
EC19	3	2	4	2	3	2	3	0	5	5	5	3	0
EC23me	3	nd	4	nd	4	3	nd	nd	3	3	3	4	2
EC19me	3	nd	3	nd	4	3	nd	nd	4	4	4	4	0

*nd* not determined

Individual retinoid-responsive genes, RAR-β and CYP26A1, responded differently with respect to temporal characteristics of activation and relative activities of the different retinoids at different doses. For CYP26A1 induction, ATRA showed the highest activity; unexpectedly, EC19 had greater CYP26A1 induction activity than EC23 at lower doses, but, in contrast to ATRA, both were ineffective at 10 μM. For RAR-β induction, both synthetic retinoids were equally effective at low doses and with greater activity than ATRA, whereas at higher doses, EC23 had the greatest activity with EC19 having much lower activity compared to ATRA.

Variability in behaviour between different short-term gene-response assays can be due to a combination of factors, particularly RARE context, RAR expression and specificity [28], and retinoid metabolism. Recent structural modelling studies have shown that the RAR-β LBD is better than other RAR LBDs at accommodating the geometrically differently substituted ring of EC19 with the carboxylic acid group in the meta-position [13]; the good activity of EC19 with respect to the induction of RAR-β suggests that this induction may be driven primarily by constitutive expression of RAR-β in these cells. This is in agreement with other studies [29] implying a dependence of RAR-β induction on RAR-β itself in SH-SY5Y cells. In the Sil-15 reporter cells, β-galactosidase activity is driven by an RAR-β RARE construct; the low activity of EC19 in this system compared to the induction of RAR-β transcripts in SH-SY5Y cells may result from differences in basal RAR-β expression between the two cell types, as this appears to be relatively lower in Sil-15 parental cells [30] compared to SH-SY5Y cells.

The genomic context of RAREs which drive marker genes, the availability of promoter-specific coregulators and retinoid-specific co-regulator interactions are also important considerations for the interpretation of retinoid activity assays. Transcriptional activation by ligand-bound RARs requires ligand-dependent conformational changes in the receptor to facilitate coactivator recruitment [31]; these may vary independently of ligand-LBD affinity such that retinoids with equivalent affinity for RAR LBDs may differ in their ability to facilitate coactivator recruitment [13]. Gene-specific induction mechanisms are also evidenced by the relatively poor activity of the synthetic retinoids on CYP26A1 induction compared to ATRA; in this respect, coregulators may be critical in driving specificity because CYP26A1 needs to respond to ATRA specifically to regulate ATRA levels. It is also possible that RARs may bind to other endogenous ligands, such as ATRA metabolites [32], and these should also be considered as potential drivers of CYP26A1 induction. Clearly, single gene assays have limited use as surrogate markers of the biological properties of synthetic retinoids.

Overall, these results imply that the assay based on Sil-15 cells, or on cells with an equivalent RARE-driven reporter, is a good short-term assay as it gave comparable results to longer-term, and more time-consuming, assays of biological responses for assessing the potency of novel synthetic retinoids. In the long-term assays, the relative induction of neurogenesis by different retinoids, as indicated by downregulation of markers of pluripotency and the upregulation of neuronal lineage markers, was comparable to the induction of RAR-α messenger RNA (mRNA) at a shorter timescale. This may imply a role for RAR-α as an initial step in the induction of neurogenesis, either via a transcriptional or non-transcriptional mechanism [33], and is supported by the different relative activities of EC19 in the induction of RAR-β and neurite extension in SH-SY5Y cells.

These studies also stress the importance of careful marker selection for retinoid assays. The transcription factor PAX6 is reported to promote neurogenesis [34], but in TERA2.cl.SP12 cells, PAX6 was upregulated substantially more in response to EC23 than ATRA, compared to the E-box transcription factor NeuroD1, implying that neuronal phenotypes may be driven to different extents by different retinoids. If neurogenesis is also regulated by the products of ATRA catabolism [9], then EC23 or comparable synthetic retinoids might have biological effects on neurogenesis that are qualitatively different to ATRA because of greater metabolic stability. As has been shown previously, EC19 does not induce neurogenesis of TERA2.cl.SP12 cells but induces an epithelial phenotype [18]; whether this is an RAR-driven process, perhaps mediated by different RAR specificities of EC19, is not clear. However, this could also result from non-specific effects such as arachidonic acid signalling as a consequence of high levels of lipophilic compounds impacting upon membrane lipids [20].

In summary, specific gene-induction assays for novel retinoids require careful interpretation as measures for their potential as modulators of neurogenesis. Changes in cell phenotype or behaviour over different temporal scales may, superficially, be simpler to interpret; nevertheless, a much better understanding and characterization of neuronal phenotypes and their regulation is badly needed to provide a framework for understanding the wider value of synthetic retinoids for regulating neuronal development.
